# Safety and effectiveness of percutaneous renal cryoablation with conscious sedation

**DOI:** 10.1080/2090598X.2020.1739382

**Published:** 2020-03-23

**Authors:** Sagar Rohitkumar Patel, Sean Francois, Tiagpaul Bhamber, Holt Evans, Kris Gaston, Stephen B. Riggs, Chris Teigland, Peter E. Clark, Ornob P. Roy

**Affiliations:** a Department of Urology, Atrium Health, Charlotte, NC, USA; b Department of Urology, University of North Carolina, Chapel Hill, NC, USA

**Keywords:** Percutaneous renal cryoablation, conscious sedation, general anaesthesia, complications, disease recurrence

## Abstract

**Objective:**

To investigate complications and treatment failure rates of percutaneous renal cryoablation (PRC) for small renal masses under local anaesthesia and conscious sedation (LACS), to assess the safety and effectiveness of this approach, as PRC is typically performed under general anaesthesia (GA).

**Patients and methods:**

We retrospectively reviewed PRC under LACS from 2003 to 2017. We analysed perioperative parameters between patients who successfully underwent PRC under LACS and patients with post-procedural complications or treatment failure (renal mass enhancement after successful intraoperative tumour ablation). Two-sided non-parametric and Fisher’s exact tests were performed to compare uncomplicated or disease-free PRC with the complication or treatment failure group, respectively.

**Results:**

A total of 100 PRCs under LACS were performed during the study period. Of these patients, six patients had at least one postoperative complication (6%), and treatment failure was diagnosed in nine patients (9%) after PRC [mean (SD) follow-up of 42.7 (26.6) months]. The procedural failure rate was 1%. No ablations were converted to GA. The mean tumour size was smaller in patients who had no complications during PRC compared to those who did, at a mean (SD) of 2.2 (0.6) cm vs 3.0 (1.0) cm (*P* = 0.039). The use of more intraoperative probes during the PRC was also associated with complications, at a mean (SD) 3.0 (1.4) vs 1.8 (0.8) (*P* = 0.021).

**Conclusions:**

PRC under LACS is an effective and safe procedural approach for managing small renal masses with low complication, treatment failure, and procedural failure rates. Larger renal masses and intraoperative use of multiple probes is associated with an increased risk of PRC complications.

**Abbreviations:**

BMI: body mass index; CCI: Charlson Comorbidity Index; GA: general anaesthesia; LACS: local anaesthesia and conscious sedation; PRC: percutaneous renal cryoablation; R.E.N.A.L.: Radius, Exophytic/Endophytic, Nearness, Anterior/Posterior, Location

## Introduction

In recent years, incidental findings of small renal masses have dramatically increased with the pervasive use of cross-sectional imaging [1,2]. For selected patients, image-guided percutaneous ablation has become a viable option for the treatment of small renal tumours [3,4]. Compared to more invasive procedures, e.g. open or laparoscopic partial nephrectomy, percutaneous renal cryoablation (PRC) greatly reduces post-surgical complications and recovery time [5]. Historically, PRC has been performed under general anaesthesia (GA) with endotracheal intubation. Given the risks associated with anaesthesia, particularly in patients with several comorbid conditions, PRC with local anaesthesia and conscious sedation (LACS) was developed as an alternative treatment modality [6,7]. Interestingly, PRC of small masses under LACS reduced the perioperative time and shortened hospital stay compared to PRC under GA [8]. However, to our knowledge, there are no published studies that analyse factors associated with procedural complications and treatment failure after PRC under LACS.

The economic and psychosocial impacts of more invasive operations are significant for patients. Even though thermal ablation has comparable outcomes to nephrectomies, complications do occur and local recurrence is higher in cryoablation [9]. Although uncommon, postoperative complications associated with renal mass ablation include haematoma formation, urine leak, bleeding risk, and infection [8,10,11]. However, current literature lacks information about complications, treatment and procedural failures rates after PRC under LACS. In the present retrospective study, we determined the safety and effectiveness of PRC under LACS by analysing postoperative complications, treatment failure, and procedural failure rates.

## Patients and methods

### Patient population

The Institutional Review Board approved the retrospective analysis of patients who underwent PRC with LACS for renal masses from 2003 to 2017. Data were obtained from Atrium Health at Carolinas Medical Center Main campus. Patients diagnosed with a solid, enhancing renal tumour underwent complete history and physical, laboratory testing, and radiological confirmation. The following variables were obtained for the study: patient demographics; past medical history; tumour features including size, laterality, polarity, location, morphology, and biopsy results; perioperative and postoperative parameters.

### Description of procedure

PRC candidates were selected by urologists, and the procedure was performed by radiologists experienced with percutaneous cryoablation. Patients were sedated with 1–6 mg midazolam (Versed) and 75–150 mg fentanyl. Lidocaine (1%) was used at the probe site for local anaesthesia. For the PRC procedure, the patient was placed prone or oblique to locate the renal mass with CT and/or ultrasonography. Before 2017, renal tumour biopsy was obtained in selected patients on a case-by-case basis. Since 2017, the practice has altered such that all patients now undergo an attempt at biopsy before PRC. At the time of the ablation, the cryoablation probe (Galil Medical Inc., St Paul, MN, USA) was inserted into the mass via CT or ultrasonographic guidance. The probe was cycled through two freeze–thaw stages: 10 min of freezing (to at least – 40°C), 8 min of active thaw, and 10 min of re-freezing followed by another thaw; no thremocoupling was performed with the ablation technique. Following the final thaw, the cryoprobe was removed. CT is performed during the procedure to determine the ice ball size and after the removal of the probe to evaluate for complications or residual mass tissue. After the PRC, patients were observed until mental status returned to baseline and discharged the same day of the procedure. If patients showed persistent altered mental status or haemodynamic instability, they were admitted overnight for observation and appropriate management.

### Postoperative follow-up and data collection

Patients were scheduled for 1-month follow-up appointments to monitor renal function via basic metabolic panel and recovery after the procedure. Following the initial postoperative outpatient appointment, patients were scheduled for regular follow-up visits between 3 and 6 months with contrast-enhanced CT imaging to assess for any residual renal tumour. Annual abdominal CT and chest X-ray were obtained at 5 years post-PRC to assess for treatment failure. Renal biopsy was scheduled if there was clinical suspicion of local recurrence for patients with no documented preoperative renal biopsy. Determination of disease recurrence was based on CT results and/or postoperative renal biopsy. Treatment failure was defined as renal mass enhancement after successful intraoperative tumour ablation. Procedural failure was defined as inability to execute PRC intraoperatively. Information about patient’s R.E.N.A.L. (Radius, Exophytic/Endophytic, Nearness, Anterior/Posterior, Location) nephrometry score, maximal tumour diameter (M), central tumour location (C), myocardial infarction history (M), and complicated diabetes history (C) [(MC)2] complication risk scores [12], and complications were recorded. Postoperative complications were graded using the Clavien–Dindo Classification system.

### Statistical analysis

We used the mean and standard deviation (SD) to represent continuous variables and proportions to represent categorical variables. Statistical analysis was completed with statistical software from the SAS Institute (Cary, NC, USA), JMP® Pro. The Shapiro–Walk test was used to determine that the dataset was not normally distributed. Thus, we performed two-sided non-parametric tests (Wilcoxon test) and Fisher’s exact tests to compare uncomplicated or disease-free PRC with the complication or treatment failure group, respectively. A *P* < 0.05 was used to determine significance of statistical tests.

## Results

### Patient demographics

During the study period, 100 renal masses underwent PRC with LACS whereas only seven patients in our database underwent ablation with GA. Patient demographics are presented in Table 1. The mean (SD) patient age was 75 (11.6) years, and 64% and 36% of the cohort were male and female, respectively. The mean (SD) body mass index (BMI) was 31 (7.7) kg/m^2^ and 33% of the patients were either active smokers or had a smoking history. The mean (SD) Charlson Comorbidity Index (CCI) was 5.5 (2.1). The mean (SD) number of probes used during the PRC was 1.9 (0.9). CT-guided ablation was performed in 98 cases, while two cases were guided by ultrasound. In all, 29% of the patients had biopsies taken during or prior to the procedure. Six patients were lost during follow-up, with a mean (SD) period from time of procedure to last follow-up appointment of 43.5 (26.7) months.

**Table 1.** Patient demographics and treatment parameters.

Variable	Value
Number of patients	100
Age, years, mean (SD)	75 (11.6)
Gender: male, female, %	64, 36
BMI, kg/m^2^, mean (SD)	31 (7.7)
Smoker, %	33
CCI Score, mean (SD)	5.5 (2.1)
Number of probes, mean (SD)	1.9 (0.9)
Sedation time, min, mean (SD)	65.6 (2.4)
Biopsy, %	29

### Tumour characteristics

Tumour characteristics are listed in Table 2. The mean (SD) total R.E.N.AL. score was 5.96 (1.9). The mean (SD) tumour size was 2.22 (0.68) cm, with 17% of patients having tumours of >3 cm. In all, 19% of tumours were located anteriorly and 49% were posterior on the kidney. Of the renal masses, 13 were in the upper pole, 41 were in the middle pole, and 37 were in the lower pole (of note, select patients’ R.E.N.A.L. score components were not documented in the electronic medical records, creating discrepancies in our total percentage calculations). Tumour biopsies were not taken in 71% of patients. Positive biopsy specimens included 18 RCCs, one oncocytoma, and one angiomyolipoma.

**Table 2.** Tumour characteristics.

Variable	Value
Total R.E.N.A.L. Score, mean (SD)	5.96 (1.9)
R Score: tumour size, cm, mean (SD)	2.22 (0.68)
Tumour >3 cm, %	17
E Score: tumour growth pattern, %	
Exophytic	53
Mesophytic	32
Endophytic	15
N Score: nearness to collecting system, %	
≥7 mm	64
4–7 mm	15
<4 mm	19
A Score: anatomical location, %	
Anterior	19
Posterior	49
Neither	32
L Score: polarity line, %	
Above/below	39
Crosses polar line	31
50% crosses pole/between poles	28
Tumour site, %	
Upper Pole	13
Middle Pole	41
Lower Pole	37

### Complication and treatment failure rates

Six patients (6%) had complications during the PRC and nine patients (9%) had treatment failure; the procedural failure rate was 1%, due to poor renal visualisation during intraoperative CT. The mean (SD) follow-up interval for the treatment failure group was 42.7 (26.6) months. None of the PRCs were converted from LACS to GA. Three patients required overnight observation for significant comorbidity, and four patients were hospitalised for more than one night for post-procedural complications. Case descriptions for patients with complications and treatment failure after PRCs are outlined in Table 3. Of the variables analysed, only tumour size and number of probes were significantly associated with complication rates (Table 4). The mean (SD) tumour size in patients who underwent PRC was larger in cases with complications than without complications, at 3.0 (1.0) vs 2.2 (0.6) cm (*P* = 0.039). Higher mean (SD) number of intraoperative probes during the cryoablation was associated with complications, at 3.0 (1.4) vs 1.8 (0.8) (*P* = 0.021). Patient’s age, age-adjusted CCI, BMI, and total R.E.N.A.L. score were not associated with PRC complications. In the complication group, there was a non-significant trend toward numerically higher CCI scores and older age compared to those without a complication (*P* = 0.118 and *P* = 0.189, respectively). Patients with complications had significantly longer hospitalisations (4.1 days) compared to these without complications (*P* < 0.001). Complications and treatment failure were not associated with (MC)2 risk scores (*P* = 0.838 and *P* = 0.356, respectively). Documented complications during the PRC under LACS included: ureteric injury, haematoma, bleeding, pneumonia, and acute renal failure. No parameters were associated with treatment failure (Table 4). Of the nine patients who had local recurrence, the options of active surveillance, re-ablation, and surgery were offered. Four patients died of non-cancer-related diseases. Five patients underwent repeat PRC. Three patients were managed with radial nephrectomy; one patient underwent partial nephrectomy. All nine patients remained disease free after secondary interventions.

**Table 3.** Parameters for patients with postoperative complications or treatment failure after PRC.

Patient no.	Tumour size, cm	Tumour location	R.E.N.A.L. Score	No. of probes	Complications (Grade)	Treatment failure	Procedural failure	CCI Score	ASA Score
1	1.8	Mesophytic left upper pole	4	-	Unable to perform	No	Yes	6	I
2	1.5	Mesophytic left lower pole	5	1	Haematoma (1)	No	No	7	III
3	2.2	Endophytic right lower pole	4	2	Ureteric Injury (3a)	No	No	5	II
4	4	Endophytic right middle pole	9	3	Pneumonia (2)	Yes	No	5	III
5	3.9	Exophytic left middle pole	9	4	Bleeding (1)	No	No	7	II
6	3.6	Mesophytic right lower pole	9	5	Haematoma, Renal failure (4a)	No	No	10	I
7	3	Endophytic right upper pole	8	3	Bleeding (1)	No	No	7	II
8	1.4	Endophytic right lower pole	6	2	None	Yes	No	3	II
9	1.6	Mesophytic right middle pole	4	1	None	Yes	No	2	III
10	1.8	Exophytic left lower pole	4	1	None	Yes	No	7	IV
11	2	Exophytic left lower pole	6	2	None	Yes	No	8	II
12	2	Exophytic left lower pole	6	2	None	Yes	No	8	II
13	2.2	Mesophytic right lower pole	5	2	None	Yes	No	8	II
14	1.8	Exophytic right lower pole	6	1	None	Yes	No	5	III
15	2.5	Exophytic left hilum	8	2	None	Yes	No	7	IV

ASA: American Society of Anesthesiologists; Grade was determined by the Clavien–Dindo Classification system.

**Table 4.** Univariate analysis of pre- and intraoperative variables for complications and treatment failure after PRC.

	Complications			Treatment failure		
Variable	No	Yes	*P*	No	Yes	*P*
No. of patients	94	6		91	9	
Gender, %						
Male	64.9	50.0	0.664	65.9	55.6	0.277
Female	35.1	50.0	0.664	34.1	44.4	0.277
Age, years, mean	74.7	80.8	0.189	75.2	74.0	0.962
Tumour size, cm, mean	2.2	3.0	0.039*	2.2	2.1	0.571
Tumour side, %						
Right	46.8	66.7	0.423	47.3	55.6	0.734
Left	53.2	33.3	0.423	52.7	44.4	0.734
Number of probes, mean	1.8	3.0	0.021*	1.9	1.8	0.802
CCI, mean	5.4	6.8	0.118	5.5	5.9	0.423
BMI, kg/m^2^, mean	31.0	29.0	0.586	30.5	34.5	0.097
R.E.N.A.L. Score, mean	5.9	7.3	0.109	6.0	6.0	0.990
(MC)2 Score, mean	3.9	4.0	0.838	3.8	4.3	0.356
Maximal tumour diameter	2.6	3.3	<0.001*	2.7	2.7	0.926
Myocardial infarction history	0.0	0.0	0.802	0.0	0.0	0.755
Central tumour location	0.1	0.3	0.368	0.1	0.0	0.363
Complicated diabetes	1.1	0.5	0.336	1.0	1.7	0.179
Hospital stay, days, mean	0.03	4.1	<0.001*	0.3	0.0	0.606

*Indicates statistical significance; note: Wilcoxon test and Fisher’s exact were performed for continuous and categorical variables, respectively.

## Discussion

The risks associated with GA cannot be disregarded. In recent years, there has been an overall rise in anaesthesia-related mortality given that surgical interventions are more common among more frail patients and the number of complex invasive operations has increased [13]. Furthermore, with older adults living longer, the geriatric population is undergoing more procedures. These procedures under GA have a higher incidence of postoperative complications such as: delirium, cognitive dysfunction, delayed rehabilitation, and mortality [14]. Given these facts, LACS for PRC has become a viable option for small renal masses. However, the PRC literature focusses on cases employing GA. We explored perioperative parameters that are associated with procedural complications and treatment failure to assess the safety and effectiveness of PRC with this novel mode of anaesthesia. Specifically, our present study showed that PRC under LACS is a viable option for complex renal tumours and patients with significant comorbidity.

Recent studies have shown that PRC with LACS is as safe and effective as GA [8]. A larger series by de Kerviler et al. [15], initially demonstrated PRC was a feasible option without GA, with low pain scores and short procedure duration, but noted a higher complication rate of 14.0%. In support, our present data shows that PRC under LACS has a lower complications rate (6%). In our present study, the treatment failure rate of 9% is similar to previously reported recurrence rates after PRC under LACS (5.8%) and GA (6%) [8,16]. Not surprisingly, patients who underwent PRC with LACS had a significantly shorter hospitalisation course (average 1.08 days) compared to GA (average 1.95 days) [8]. With comparable surgical outcomes to ablation with GA, PRC under LACS may confer advantages for selected patients given the risks associated with anaesthesia. As LACS does not necessarily require consultation of the anaesthesiology team compared to GA, this may alleviate healthcare expenses. Cost–benefit analysis of several other minimally invasive procedures also favour the use of LACS over GA [16]. Further investigation is necessary to quantify the cost benefit of PRC with LACS compared to other forms of sedation, especially as the procedural failure rate is extremely low (1%) and no PRCs were converted to GA.

The R.E.N.A.L. nephrometry score is associated with more complications in PRC with GA [17]. Our present study is the first to assess the association of R.E.N.A.L. nephrometry score with postoperative complications and ablation failure under LACS, suggesting that these rates are not associated with more complex renal masses. The (MC)2 PRC risk calculator, developed from PRC under GA, was refuted by analysis of our present cohort with LACS [18]. Moreover, our present data demonstrated that tumour size was significantly larger in those patients who had complications. In a recent study, the most important factor in predicting major PRC complications was tumour diameter for patients under GA and often associated with post-procedural bleeding [18]. Our present data is consistent with the Schmit et al. [18] study, in that tumour diameter is predictive of postoperative complications in both types of anaesthesia [4]. Furthermore, higher BMI and comorbidity did not increase the risk of post-procedural injury during LACS in our present study. Other than tumour bulk, PRC under LACS is relatively safe in regard to tumour complexity and comorbidity status.

Post-procedural bleeding is the most common complication after PRC. Studies note that 0.9–8.3% of PRC cases require blood transfusion [12]. In our present study, two patients had postoperative bleeding (2%); both these cases were able to be managed conservatively without the need for transfusions or embolisation. These patients had a tumour burden of 3 and 3.9 cm, which has been shown to be a risk factor for postoperative bleeding [19].

Similar to postoperative bleeding, the incidence of haematoma formation significantly increases with increasing tumour size and the number of probes used during the procedure [10]. Of the perirenal haematoma complications, only one probe was used in one of the patients. Hafron and Kaouk [12] stated that haemorrhage was solely associated with the use of multiple probes. The average number of probes used during PRC without complications was 1.8 probes in our present study. Our present data demonstrates that significantly more probes (mean 3.0) were used intraoperatively for patients that subsequently developed complications.

Injuries to the collecting system have been previously reported after PRC [20]. Sung et al. [21] studied the short- and long-term sequelae of intentional cryoablation of the renal collecting system. Collecting system injury is fairly uncommon; often these injuries heal by secondary closure with stent placement [22]. In our present retrospective analysis, we reported one ureteric complication requiring JJ-stent placement demonstrating the low rate of ureteric injury.

There are several limitations to the present study including small sample size, lack of comparison group with GA, short duration of follow-up and low events of complications. Due to retrospective analysis, data extrapolated from the study can only show associations and is prone to selection bias. Only 29% of the cohort had biopsies. For the remaining patients, we are uncertain about the pathology of the renal tumour, limiting the extent to which we can evaluate effectiveness of PRC for certain types of renal masses. Prior clinicians at our hospital system did not routinely perform renal biopsies before PRC because of the perceived increased risk of complications from the biopsy itself. However, with more experience and recognition of the safety and benefits of percutaneous renal mass biopsy, radiologists at our hospital have started to routinely collect pathology specimens before ablation. Despite these limitations, we demonstrate that PRC under LACS can be performed safely in patients with complex renal tumours and significant comorbidity, with relatively low post-procedural complications, re-treatment, and procedural failure rates.

## Conclusion

PRC under LACS is an efficacious and safe procedure for managing small renal masses with a low complication and treatment failure rate, similar to that seen in series using GA. Larger renal masses and intraoperative utility of multiple probes increases the risk of PRC complications. With this knowledge, clinicians can better select patients for renal tumour ablation and educate patients about PRC under LACS, as well as consistently trust this technique due to low procedural failure and conversion to GA rates.
